# Viral Chromosome Conformation Capture (V3C) Assays for Identifying Trans-interaction Sites between Lytic Viruses and the Cellular Genome

**DOI:** 10.21769/BioProtoc.3198

**Published:** 2019-03-20

**Authors:** Kinjal Majumder, Maria Boftsi, David J Pintel

**Affiliations:** 1 Department of Molecular Microbiology and Immunology, University of Missouri School of Medicine, Columbia, Missouri, USA; 2 Christopher S. Bond Life Sciences Center, University of Missouri-Columbia, Columbia, Missouri, USA; 3 Pathobiology Area Graduate Program, University of Missouri-Columbia, Columbia, Missouri, USA

**Keywords:** Chromosome conformation capture, Parvovirus, DNA damage response, Topologically associating domains, Fragile sites, Host-pathogen genome interactions

## Abstract

The folding mechanisms of the mammalian genome package our genetic material into the nucleus, and in doing so, dictate its appropriate replication and expression. Chromosome conformation capture technology has enabled the dissection of the folding principles of the cellular genome. This has led to a better understanding of the role played by architectural proteins in forming and dissolving 3D-chromatin-structure. These assays are based on the principle of crosslinking distant cellular sites that are proximal to each other in 3D space using formaldehyde followed by digestion of formed hybrid DNA junctions. Invading viruses, such as the lytic parvovirus Minute Virus of Mice (MVM), establish distinct replication centers within the nuclear environment at cellular sites that preferentially undergo DNA damage, but do not integrate into the cellular DNA. We have adapted chromosome conformation capture technology to study the trans-interaction between MVM and the cellular genome, which we have dubbed V3C, which can be extended to a whole-genome analysis we term V3C-seq. This protocol describes the procedure for performing, as well as analyzing V3C-seq assays, and can be adapted for mapping the cellular interaction sites of any non-integrating DNA virus.

## Background

**Figure 1.**
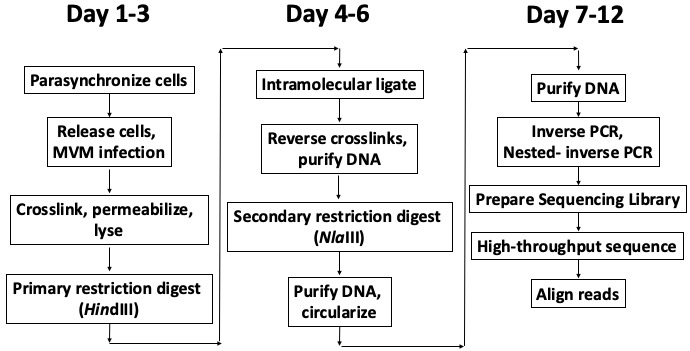
Schematic of the V3C-seq assay with approximate timeline

Chromosome conformation capture technologies have helped us gain significant insights into the folding principles of the mammalian genome, helping identify the short-range promoter-enhancer loops that underlie epigenetic regulation of gene expression, as well as long-range multi-loop domains that form sub-chromosomal chromatin compartments (Dekker *et al.*, 2013; Denker and de Laat, 2016). While these technologies have helped define important rules related to the patterns of cis-folding of the cellular genome, their implementation in studying the trans-interaction between the genome of an invading DNA virus and its target cell remained largely unstudied. In this protocol, we describe the procedure for studying the interactome of the parvovirus Minute Virus of Mice (Figure 1). Owing to its 5 kilobase genome, the application of a modified Circular Chromosome Conformation Capture (4C) assay combined with high-throughput sequencing (Van De Werken *et al.*, 2012) allowed the generation of a high resolution map of the sites on cellular DNA where MVM localizes during infection (Majumder *et al.*, 2018).

## Materials and Reagents

1. Pipette tips (Fisher, catalog numbers: 02-707-426, 02-707-403, 02-707-438)
2. 15 ml falcon tubes (Thermo Scientific, catalog number: 339650)
3. 50 ml falcon tubes (Thermo Scientific, catalog number: 339652)
4. Eppendorf tubes (Fisher, catalog number: 05-408-129)
5. Stericup-GP Sterile Vacuum Filtration System (Millipore, catalog number: SCGPU11RE)
6. Disposable Cell Scrapers (Fisher, catalog number: 08-100-241)
7. Murine A9 fibroblasts
8. Human NB324K kidney cells
9. MVMp virus
10. Primers (IDT)
  Inverse PCR primer:
  F: 5’-gaggcaaggttggtcactactt-3’
  R: 5’-caggaactttgccccattta-3’
  Nested Inverse PCR primer sequences:
  F: 5’-ggcaaggttggtcactactttt-3’
  R: 5’-gccccatttagcacagtagc-3’
Primers for determining *Hin*dIII digestion efficiency:
  P1: 5’-agaaaattggcatcggtttg-3’
  P2: 5’-tgactgtagctcctccaaattgt-3’
  P3: 5’-ttttggcaggtgtccttttc-3’
11. iTaq Universal SYBR Green Supermix (Bio-Rad, catalog number: 1725121)
12. liquid nitrogen (Airgas Mid-America)
13. 37% formaldehyde (Fisher, catalog number: F79-4)
14. 1 M glycine (Fisher, catalog number: BP381-5)
15. Protease Inhibitor Cocktail (MedChemExpress, catalog number: HY-K0010)
16. Sodium Dodecyl Sulfate (SDS; Fisher, catalog number: BP166-500)
17. Triton X-100 (Fisher, catalog number: BP151-100)
18. Proteinase K (Thermo Scientific, catalog number: AM2542)
19. RNase A (Sigma-Aldrich, catalog number: R4875-500MG)
20. Glycogen (Sigma-Aldrich, catalog number: 10901393001)
21. Isopropanol (Fisher, catalog number: A451SK-4)
22. 70% ethanol (Fisher, catalog number: AC615090010)
23. Buffer EB (QIAGEN, catalog number: 19086)
24. PCR Purification Kit (QIAGEN, catalog number: 28106)
25. *Nla*III (NEB, catalog number: R0125L) (store at -80 °C)
26. *Hin*dIII (NEB, catalog number: R0104L)
27. 10x NEB Restriction Enzyme buffer 2.1, Cutsmart buffer (supplied with *Hind*III and *Nla*III enzymes; see above for catalog numbers)
28. T4 DNA Ligase (NEB, catalog number: M0202L)
29. 10x NEB T4 DNA Ligase buffer (NEB, catalog number: B0202S)
30. Platinum Taq DNA Polymerase High Fidelity (Thermo Fisher, catalog number: 11304029)
31. NEBNext Ultra II DNA Library Prep Kit for Illumina (NEB, catalog number: E7645S)
32. DMEM (Gibco, catalog number: 11965-092)
33. L-glut (Fisher, catalog number: BP379-100)
34. Fetal Calf Serum (FCS) (Fisher, catalog number: SH30073.03)
35. Gentamicin (Fresenius Kabi USA , catalog number: 63323-010-20)
36. Phosphate Buffered Saline (Fisher, catalog number: SH3025601)
37. Tris-base (Fisher, catalog number: BP152-5)
38. Sodium Chloride (NaCl) (Fisher, catalog number: BP358-10)
39. Calcium Chloride (Sigma-Aldrich, catalog number: C7902-500G)
40. Potassium Chloride (Fisher, catalog number: BP366-500)
41. Magnesium Sulfate (MgSO_4_) (Fisher, catalog number: BP213-1)
42. Sodium Dihydrogen Phosphate (Amresco, catalog number: 0571-500G)
43. Glucose (Fisher, catalog number: D16-1)
44. NP40 (Sigma-Aldrich, catalog number: CA-630)
45. L-arginine (Sigma-Aldrich, catalog number: A8094-25G)
46. L-cysteine (Sigma-Aldrich, catalog number: C7602-25G)
47. L-histidine (Sigma-Aldrich, catalog number: H6034-25G)
48. L-leucine (Sigma-Aldrich, catalog number: L8912-25G)
49. L-lysine (Sigma-Aldrich, catalog number: L8662-25G)
50. L-methionine (Sigma-Aldrich, catalog number: M5308-25G)
51. L-phenylalanine (Sigma-Aldrich, catalog number: P5482-25G)
52. L-threonine (Sigma-Aldrich, catalog number: T8441-25G)
53. L-tryptophan (Sigma-Aldrich, catalog number: T8941-25G)
54. L-tyrosine (Sigma-Aldrich, catalog number: T8566-25G)
55. L-valine (Sigma-Aldrich, catalog number: V0513-25G)
56. 100x MEM vitamin Solution (Thermo Fisher, catalog number: 11120052)
57. HEPES (Fisher, catalog number: BP410-500)
58. sodium bicarbonate (Fisher, catalog number: BP328-500)
59. EDTA (Fisher, catalog number: S311-3)
60. 1x Phenol:Chloroform:Isoamyl alcohol (Fisher, catalog number: BP1752I-400)
61. Chloroform (Fisher, catalog number: C606SK-4)
62. Phenol red
63. TE buffer (see Recipes)
64. complete DMEM media (see Recipes)
65. 3C Lysis buffer (see Recipes)
66. Earle’s 10x salts (see Recipes)
67. Isoleucine minus media (see Recipes)

*Note: The Eppendorf tubes and Falcon tubes used in this protocol should be chloroform resistant.*

## Equipment

1. Micro-Pipettes (P2, P20, P200, P1000)
2. Biorad T100 Thermal Cycler (Bio-Rad, catalog number: 1861096)
3. Illumina Nextseq 500 (Illumina, catalog number: SY-415-1001)
4. Bellco Rocker Platform (Marshall Scientific)
5. Vortex Genie 2 (Scientific Industries Inc., catalog number: SI-0236)
6. refrigerated centrifuge (Eppendorf, catalog number: 5417C)
-80 °C freezer
-20 °C freezer
9. 4 °C refrigerator
10. 37 °C shaker (Eppendorf Thermomixer, catalog number: 05-400-200)
11. Nanodrop spectrophotometer (Thermo Fisher, catalog number: ND-ONE-W)
12. Vacuum manifold (for aspirating supernatants)
13. CFX Connect Real-Time PCR Detection System (Bio-Rad, catalog number: 1855200)

## Software

1. Bowtie 2 (Langmead and Salzberg, 2012)
2. Samtools (Li *et al.*, 2009)
3. Bedtools (Quinlan and Hall, 2010)

## Procedure

- Parasynchronization and infection of cells
  1. Plate 5 x 10^6^ cells at a concentration of 0.5 x 10^6^ cells/ml in 10 ml Isoleucine-minus media in 15 cm dishes for 36-42 h at 37 °C and 5% CO_2_. Murine A9 fibroblasts and human NB324K kidney cells are permissive to MVMp infection and can be parasynchronized by isoleucine deprivation.
  2. Release cells into G1/S-phase by aspirating the media and adding 10 ml complete DMEM media. Simultaneously, infect with MVMp virus at an MOI of 5 (25 µl of MVMp at 1 x 10^6^ Pfu/µl) for 16 h.
*Note: MVMp replication starts as cells enter into S-phase, approximately 10 h post-release into complete DMEM.*
- Crosslinking and lysis
  1. Crosslink cells in the dish by adding 540 µl of 37% formaldehyde (final concentration of 2%). Mix well by pipetting and incubate at room temperature for 10 min on a rocker (12 oscillations/min).
  2. Quench the crosslinking reaction by adding 1.425 ml of 1 M glycine (final concentration of 0.125 M). Mix well by pipetting and incubate at room temperature for 5 min on a rocker (12 oscillations/min).
  3. Gently scrape cells using a disposable cell scraper (by moving horizontally, and vertically) and collect the cell suspension (in 12 ml media, formaldehyde and glycine) in 15 ml falcon tubes.
  4. Centrifuge at 500 *× g* for 5 min at 4 °C in a refrigerated centrifuge. Aspirate supernatant with a vacuum manifold or gently with a pipette.
  5. Wash cells with 10 ml PBS.
  6. Centrifuge at 500 *× g* for 5 min at 4 °C in a refrigerated centrifuge. Aspirate supernatant.
  7. Suspend pellet in 5 ml cold 3C Lysis buffer (freshly supplemented with protease inhibitors) and incubate on ice for 10 min.
  8. Centrifuge at 3,000 *× g* for 5 min at 4 °C. Aspirate the supernatant. A small white pellet should be visible at the bottom of the tube. This is mostly made up of cell nuclei. The tube can be snap frozen in liquid nitrogen at this point and stored at -80 °C. Thaw samples on ice if starting from this step.
*Note: Aspiration should be performed using a vacuum manifold. In its absence, pipettors may be used to carefully remove the supernatants without disturbing the cell (or nuclear) pellet.*
- Primary restriction digest
  1. Suspend nuclei in 500 µl 1.2x NEB buffer 2.1 (60 µl of 10x stock diluted in 440 µl ddH_2_O) and transfer to Eppendorf tube.
  2. Add 7.5 µl of 20% SDS (final concentration of 0.3%) and place tube in a shaker at 37 °C at 900 rpm.
*Note: this process permeabilizes the nuclei. However, the formation of bubbles or too much SDS affects enzyme activity. Therefore, avoid forming bubbles, and ensure proper concentration of SDS solution.*
  3. Incubate for 1 h at 37 °C while shaking at 900 rpm.
  4. Add 50 µl of 20% Triton X-100 (final concentration of 2%).
*Note: Prepare 20% Triton solution well in advance, mix and keep at 56 °C to ensure homogeneous solution. Otherwise, SDS is inadequately sequestered, leading to premature inactivation of the restriction enzyme.*
  5. Incubate for 1 h at 37 °C while shaking at 900 rpm.
  6. Take out 10 µl aliquot as undigested gDNA Control. Store at -20 °C.
  7. Add 20 µl (400 U) of *Hin*dIII to remaining sample and incubate overnight at 37 °C while shaking at 900 rpm.
  8. Add 15 µl (300 U) of additional *Hin*dIII and incubate on a 37 °C shaker at 900 rpm for 4 h.
  9. Take out 10 µl aliquot as digested DNA Control. Store at -20 °C.
  10. Reverse crosslinks and determine digestion efficiency as follows:
    - Add 2 µl of Proteinase K to the undigested DNA and digested DNA tubes, and incubate at 56 °C for 1 h.
    - Dilute the samples to 100 µl with 90 µl ddH_2_O.
    - Add 100 µl of phenol:chloroform:isoamyl alcohol (25:24:1) and vortex (setting: 10) for 10 s.
    - Centrifuge at 13,000 *× g* at room temperature for 5 min.
    - Transfer supernatant to new Eppendorf tube and add 1 ml isopropanol, and 2 µl glycogen. Vortex thoroughly for 10 s.
    - Centrifuge at 13,000 *× g* at room temperature for 5 min and aspirate the supernatant. A white pellet should be visible at the bottom of the tube.
    - Add 1 ml of 70% ethanol to the tube.
    - Centrifuge at 13,000 *× g* at room temperature for 5 min and aspirate the supernatant.
    - Resuspend pellet in 20 µl Buffer EB.
    - Perform qPCR (using Bio-Rad SYBR Green Supermix; annealing temp: 60 °C) with 2 µl of undigested and digested gDNA using the primer sets P1-P2 (Input) and P1-P3 (undigested). Set up each qPCR reaction in triplicate.
    - Digestion efficiency is calculated from the mean Cq value of each reaction in the digested gDNA sample using the formula:
      $$Percent\;digestion=(1-2^{\left(C_{q}^{P1P2}-C_{q}^{P1P3}\right)})*100$$
- Ligation
  1. Add 40 µl of 20% SDS (final concentration of 1.6%) to the digested chromatin.
  2. Incubate for 20-25 min at 65 °C.
  3. Transfer digested nuclei to a 50 ml falcon tube.
  4. Add 6.125 ml of 1.15x Ligation buffer (10x concentrated buffer is diluted in ddH_2_O).
  5. Add 375 µl of 20% Triton X-100 (final concentration of 1%).
  6. Incubate for 1 h at 37 °C while shaking gently.
  7. Add 1 µl T4 DNA Ligase (100 U total) and incubate for 4 h at room temperature.
*Note: can also perform the ligation reaction at 16 °C.*
  8. Add 15 µl of 20 mg/ml Proteinase K (final 300 μg).
  9. Incubate at 65 °C overnight to reverse crosslinks.
*Note: We also de-crosslink at 56 °C. Any temperature within this range reverses protein crosslinks without affecting the DNA.*
- DNA purification
  1. Add 10 µl of 10 mg/ml RNase A (final 100 µg).
  2. Incubate for 30-45 min at 37 °C.
  3. Add 7 ml phenol-chloroform and mix by vortexing (setting: 10).
  4. Centrifuge for 5 min at 2,000 *× g* at 4 °C.
  5. Collect the upper aqueous phase, transfer to a new tube, add 7 ml chloroform, vortex (setting: 10).
  6. Centrifuge for 5 min at 2,000 *× g* at 4 °C.
  7. Transfer supernatant into a 50 ml tube, add 25 ml isopropanol and 5 µl glycogen (10 mg/ml).
  8. Mix and place at -80 °C for 1 h.
  9. Centrifuge for 20 min at 4,200 *× g* at room temperature.
  10. Remove supernatant and add 10 ml 70% ethanol.
  11. Centrifuge for 15 min at 4,200 *× g* at 4 °C.
  12. Remove supernatant and dry pellet at room temperature.
  13. Dissolve DNA pellet in 200 µl of Buffer EB.
- Secondary restriction digest
  1. Set up *Nla*III digest on all of the 3C DNA overnight at 37 °C with 25 µl of 10x Cutsmart buffer and 15 µl of *Nla*III.
  2. Inactivate *Nla*III by incubating at 65 °C for 20 min.
  3. Add 1x Phenol:Chloroform:Isoamyl alcohol (250 µl); vortex, spin (10,000 *× g*), collect supernatant.
  4. Add 250 µl chloroform, vortex, spin (10,000 *× g*), collect supernatant.
  5. Add 700 µl of isopropanol and 2 µl glycogen (10 mg/ml), vortex, spin (10,000 *× g* to precipitate), aspirate supernatant.
  6. Add 1 ml of 70% ethanol, spin (10,000 *× g*), aspirate supernatant.
  7. Resuspend in 1 ml of ultrapure water.
- Circularization
  1. Set up ligation reaction overnight with 1.5 ml of 10x ligase buffer, 12.5 ml of water, 1 ml resuspended *Nla*III digested DNA, 1 µl T4 DNA ligase in a 50 ml Falcon tube.
  2. Add 15 ml Phenol:chloroform:Isoamyl alcohol, vortex, spin (4,200 *× g*), collect supernatant.
  3. Add 15 ml Chloroform, vortex, spin (4,200 *× g*), collect supernatant.
  4. Add 25 ml Isopropanol and 5 µl glycogen (10mg/ml) to precipitate DNA. Spin at 4,200 *× g*.
  5. Wash with 10 ml of 70% ethanol; spin at 4,200 *× g* and aspirate supernatant.
  6. Air dry the pellet at room temperature for 20 min.
  7. Resuspend in 200 µl Buffer EB.
  8. Purify with QIAGEN PCR purification kit and elute twice in 100 µl buffer EB.
- Inverse PCR
  1. Quantify V3C-seq product by Nanodrop and load 200-600 ng of template DNA per PCR.
  2. Set up inverse PCR using Platinum Taq DNA Polymerase High Fidelity from Thermo Scientific as follows:
    Template DNA  2 µl
    10x PCR buffer     2.5 µl
MgSO_4_ 1 µl
    dNTP 0.5 µl
    Primer 1 0.5 µl
    Primer 2 0.5 µl
    HIFI Taq 0.1 µl
ddH_2_O 17.9 µl
  3. Inverse PCR reaction conditions
    94 °C for 2 min

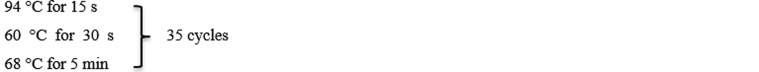

    68 °C for 10 min
    4 °C (hold)
  4. Dilute PCR product 1:100 in TE buffer.
  5. Set up nested inverse PCR reactions with diluted PCR product as template.
  6. Purify nested inverse PCR product with PCR purification kit (QIAGEN) and elute in 50 µl Buffer EB.
  7. Nanodrop inverse PCR product and generate sequencing library with NEBNext Ultra II DNA Library Prep Kit for Illumina.
  8. Pool up to 12 samples per lane and sequence samples by high throughput sequencing using the Illumina NextSeq 500 platform.

## Data analysis

1. Align the sequencing reads using Bowtie 2
  - module load bowtie2/bowtie2-2.2.9
  - bowtie 2 --trim5 20 --very-sensitive -x mm10 -S mvm_1.sam mvm_1.fastq
2. Convert .sam files into .bam files and sort .bam files using Samtools
  - module load samtools/samtools-1.3.1
  - samtools view -b -S -o mvm_1.sam > aligned_mvm_1.bam
  - samtools sort -o aligned_mvm_1.bam sorted_aligned_mvm_1.bam
3. Compute histograms using BEDtools
  - module load bedtools/bedtools-2.26.0
  - genomeCoverageBed -ibam sorted_aligned_mvm_1.bam -bg -trackline -split -g ... > mvm_1.bedgraph
4. Upload bedgraph file to UCSC genome browser
  In the “edit configuration” field, type: track type=bedGraph name="mvm_1" description="mvm_1" priority=20
A representative histogram of MVM interaction sites on mouse chromosome 17 is shown in Figure 2B (top panel). These sequencing tracks can be compared with ChIP-seq data, such as the DNA damage marker gamma-H2AX during MVM infection of A9 cells (shown in the bottom panel of Figure 2B).
5. The MVM interaction sites identified by V3C-seq can be validated using focused Taqman-based 3C-qPCR assays, such as the validation of MVM interaction with the 5’ end of mouse chromosome 19 (Figure 2A, left column). In order to account for background interactions, these assays should include controls where the crosslinks are reversed prior to ligation (Figure 2A, middle column) and unligated controls (Figure 2A, right column). More information on primers and probes used for the 3C-qPCR assays can be found in Majumder *et al.*, 2018.
  **Figure 2.**
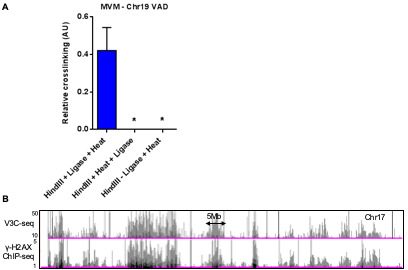
quantification of MVM-host hybrid DNA formation using focused qPCR and high-throughput sequencing assays. A. Taqman qPCR analysis of MVM interaction with a cellular site on mouse chromosome 19, with the probe sequence complementary to the MVM genome. This control experiment shows that reversing the crosslinks prior to ligation (middle) or excluding the intramolecular ligation step (right) prevents the formation of virus-host hybrid junctions. The figure has been replotted from experiments published inMajumder *et al.* (2018). B. Representative histogram on chromosome 17 of V3C-seq in MVM infected murine A9 fibroblasts with the viewpoint on the 5’ end of the MVM genome (top). The V3C-seq data can be compared with other high-throughput sequencing experiments. As an example, ChIP-seq for the DNA damage associated chromatin mark gamma-H2AX in MVM infected murine A9 cells at 20 h post infection is shown on the track below. For more details, refer toMajumder *et al.* (2018) and publicly available V3C-seq and ChIP-seq data in the GEO repository (Accession number GSE112957).

## Recipes

1. TE Buffer
  10 mM Tris-HCl, pH 8.0
  1 mM EDTA, pH 8.0
2. complete DMEM media (500 ml)
  DMEM (with L-glut)
  5% FCS
  500 µl Gentamicin (50 mg/ml)
3. 3C Lysis buffer
  10 mM Tris-HCl, pH 7.5
  10 mM NaCl
  0.2% NP40
4. Earle’s 10x Salts (1L)
  2.645 g Calcium Chloride
  4 g Potassium Chloride
  2 g Magnesium Sulfate
  68 g Sodium Chloride
  1.22 g Sodium Dihydrogen Phosphate
  10 g Glucose
  0.1 g Phenol red
5. Isoleucine minus media
Add in order ~350 ml ddH_2_O
  50 ml Earle’s 10x salts
  0.5 ml 1,000x L-arginine
  0.5 ml 1,000x L-cysteine
  0.5 ml 1,000x L-histidine
  0.5 ml 1,000x L-leucine
  0.5 ml 1,000x L-lysine
  0.5 ml 1,000x L-methionine
  0.5 ml 1,000x L-phenylalanine
  0.5 ml 1,000x L-threonine
  0.5 ml 1,000x L-tryptophan
  0.5 ml 1,000x L-tyrosine
  0.5 ml 1,000x L-valine
  0.5 ml 100x vitamins
  600 µl Gentamicin
  5 ml 1 M HEPES, pH 7.5
Adjust pH with 7.5% sodium bicarbonate (until red). Bring up to 475 ml with ddH_2_O. Filter (0.22 μm) sterilize and dialyze against 5% serum. Store at 4 °C
